# Family Group Conferences within the integrated care system for young people with ID: a controlled study of effects and costs

**DOI:** 10.1186/s12913-015-1062-2

**Published:** 2015-09-18

**Authors:** Simone A. Onrust, Geke Romijn, Yvette de Beer

**Affiliations:** Trimbos Institute, PO Box 725, 3500 AS Utrecht, The Netherlands; VU University, De Boelelaan 1105, 1081 HV Amsterdam, The Netherlands; Expertisecentrum William Schrikker, PO Box 12685, 1100 AR Amsterdam, The Netherlands

## Abstract

**Background:**

The Dutch healthcare system and the roles of the government and citizens are changing. The government will be limiting its role in care and assistance, while citizens will be expected to increasingly care for themselves and each other. An important instrument to support this transformation involves utilizing people’s social network, in the form of the Family Group Conference. Studies on the use of these Family Group Conferences within various sectors are promising. Whether the Family Group Conference is also effective within the integrated care system for young people with intellectual disability (ID) is not yet known.

**Methods:**

In this study, anonymized file data were collected from 71 clients who had taken part in a Family Group Conference and a comparable group of 53 clients who had not. Information about the present areas of concern in the family was retrospectively collected and scored by means of a standardized protocol. In addition, information about received care and support from the integrated care system for young people with ID was collected. The areas of concern were assessed at two moments in time, with a 12-month interval. Resource use was assessed for the entire research period of 12 months.

**Results:**

The problems in the group of clients who had taken part in a Family Group Conference greatly decreased over a period of twelve months. There was a much smaller decrease in the number of problems in the group that had not taken part in a Family Group Conference. Resource use did not significantly differ between conditions.

**Conclusions:**

Our findings reveal that people with ID can also benefit from this approach, something which had been previously doubted. Support from the social network, however, does not substitute formal care.

## Background

In The Netherlands, the government has always played an important role in the care for people with a wide range of needs. For example, Dutch law states that parents and children with parenting or developmental problems have the right to receive government-funded care. People with intellectual disability (ID) have the right to receive care under the Exceptional Medical Expenses Act, also funded and organized by the government.

Most countries define people with ID as those with an IQ below 70. In the Netherlands, however, people with an IQ between 70 and 85 are also considered as having ID if they have severe problems of adaptive behaviour [1]. These people are known to be often in need of long-term assistance [2]. People with ID in the Netherlands have access to various specialized forms of care, such as special education, special work-study programs and specialized care for both children and adults with disability.

However, it is uncertain whether this will remain the case in the future. In recent years it has become clear that on the one hand the costs of the healthcare system are increasing dramatically, and on the other hand that citizens are not given enough opportunities to take initiatives or to provide their own solutions. The result is a change in the Dutch healthcare system and the roles of the government and citizens. There is a tendency towards increasing autonomy, self-reliance, self-efficacy and taking responsibility for one’s own life. The government will be limiting its role in care and assistance, while citizens will be expected to increasingly care for themselves and each other. Citizens will get the opportunity to take their own initiatives while the government will focus on providing care for those who are considered most vulnerable.

This societal development directly influences the nature of healthcare provision. While healthcare was previously supply-driven, with a problem-focused orientation, it is now often centred around the client’s request for help and provided from a solution-focused orientation. There is a shift from a focus on problems and concerns to a focus on strengths and solution-oriented thinking. Attention for opportunities and capabilities of citizens is growing. Realistic solutions are pursued that can prevent the problems from recurring in the future.

An important instrument to support this transformation involves utilizing people’s social network, in the form of the Family Group Conference (or family-group decision making). Such a Family Group Conference uses a decision-making model in which a plan is developed by the family and their social network. For this purpose, a special meeting is organized, a so-called conference or deliberation. A coordinator or social worker supports the family in organizing the meeting, but the family may decide who will be invited to it. The participants could be relatives, but friends, neighbours or other people trusted by the family may be included as well.

In recent years a lot of research has been done on the use of these Family Group Conferences within various sectors. At first this mainly concerned process evaluations and exploratory research, but in the past few years a number of controlled studies have been conducted [3–6]. Studies without a control group mainly showed positive effects, such as increased cohesion within the network, improvements to the child’s situation, activation of the social network, a shift of control to the family, improved relationships between the family and the social worker, and a decrease in healthcare usage [7–9]. In New Zealand, where the Family Group Conference is a citizen’s legal right, the number of children placed under supervision by the government has decreased by 60 % [10]. Results of controlled studies are less unequivocal. For instance, Sundell and Vinnerljung [3] found various negative results, including longer placements into court custody, in comparison with the control group, whereas Wijnen-Lunenberg et al. [5] and Pennell et al. [6] found quicker improvements and shorter stays in institutions.

Not much research has been conducted on the effect of the Family Group Conference within the integrated care system for young people with ID. The available, mostly unpublished, research is mainly of a qualitative nature and focuses more on the suitability and degree of satisfaction. While most of such research indicates that the Family Group Conference also seems to be suitable within this integrated care system, many social workers have considerable doubts about this.

These doubts arise from the main characteristics of ID, such as social awkwardness, a weak sense of social responsibility, impaired social skills and a higher rate of problems of general social functioning [11]. The result is often a very limited social network and an isolated life, which reduces the benefits people can derive from their social network [12, 13].

Furthermore, 50 % of young people with ID have severe emotional and behavioural problems [14]. Many of these youngsters come from multi-problem families, which are characterized by limited self-efficacy, dysfunctioning and accumulating problems, such as financial problems, low socioeconomic status, parents with addiction or mental problems, or divorced parents [11, 15–17]. People with ID who are also having behavioural, parenting or criminal behaviour problems often have a social network with similar problems. As a result, using the social network might actually have a negative effect on the development of the current problems.

On the other hand, there are convincing arguments to examine the effect of empowering children or parents with ID. After all, each citizen has the right to first make their own plans before the government (or professionals working for the government) intervenes in the lives of young people. This enables citizens to remain in control and take responsibility for their own lives. The basic assumption is that people themselves are better able to find effective solutions to their problems than a professional. In addition, they are more motivated than the professionals to find permanent solutions. Various studies have found that involving children and young people in the decision-making process results in better decisions [18].

The above-mentioned considerations induced us to conduct a controlled study into the effects of the Family Group Conference, as one of the most commonly used forms of family group decision making, on the problems of children receiving support from various organizations for children and/or parents with ID. In addition, we conducted a cost-effectiveness analyses to explore whether the Family Group Conference leads to a decrease in the use of formal care and services provided by the Dutch integrated care system for young people with ID.

## Methods

### Study sample

This study assessed the effects of Family Group Conferences within the integrated care system for young people with mild ID in the Dutch province of Overijssel. This system consists of both freely accessible facilities and providers of indicated care for children and/or parents with mild ID. Five organisations participated in this study: two freely accessible local disability support centres, two specialized youth care services offering intensive ambulatory care, specialized pedagogical support at home, semi-residential care, residential care and foster care, and a Child Protection Agency responsible for family supervision and guardianship. The participants included children with ID as well as children of parents with ID.

The intervention group consisted of all clients from the Overijssel integrated care system who had taken part in a Family Group Conference in 2011 or 2012, and whose files provided information about the family’s areas of concern at two moments in time (before the Family Group Conference and about 12 months after it). Accessibility of file data was necessary to allow us to draw conclusions about the benefits of Family Group Conferences.

The control group consisted of comparable clients who had not taken part in a Family Group Conference. The control group did not include clients refusing to take part in a Family Group Conference. In order to obtain a comparable control group, we collected file data originating from the year prior to the implementation of Family Group Conferences in the integrated care system for young people with ID in Overijssel. The control group was assembled using two methods. We first tried to find matched clients. To this end, a summary was made of the most important demographic characteristics and areas of concern of the clients of the intervention group. Next, the staff of the participating organizations were asked to look for comparable clients among their caseload. As this procedure did not result in enough files, the control group was eventually supplemented with a number of randomly selected files.

### Intervention

Although the decision-making model of Family Group Conference originates from New Zealand, these days it is being applied in many different countries. The core concept of the Family Group Conference is to help families draw up a plan together with their social network, in order to solve their problems.

In the Netherlands, Family Group Conferences are implemented by a national institution, the Family Group Conference Agency. Family Group Conferences were introduced in the Netherlands in 2000, and since then the applicants rapidly increased. During the research period, a total of 1924 clients were referred to the Family Group Conference Agency [19]. Most commonly, public or private child welfare agency social workers refer families to the Family Group Conference Agency, although some families may also self-refer. Families are usually referred for a combination of multiple problems. On average, 3,9 problems per referred family are reported to the Family Group Conference Agency. The main reasons for referral are insufficient parenting skills or neglect (63 %), behavioural problems of the child (50 %), parental stress (42 %), divorce (41 %), and financial problems (28 %) [19]. The Family Group Conference Agency utilized an application form, on which the referring worker or family member can state the questions they would like to discuss with the extended family. These questions usually refer to parenting practices (57 %), living arrangements (46 %), improvement of the child’s behaviour (42 %), improvement of the parent–child relationship (41 %), and arrangements concerning parental access (28 %) [19].

The Family Group Conference is assisted by an independent coordinator, who is not part of the social network or the healthcare institutions involved in the case. This coordinator is a trained volunteer of the Family Group Conference Agency, who organizes the conference, but is not responsible for the plan itself. In the Netherlands, there are over 600 coordinators, who all received six days of training by the Family Group Conference Agency. After referral, the coordinator will contact the family to explain the concept of the Family Group Conference. If the family agrees to participate in the Family Group Conference, the coordinator will start with the preparations for the Family Group Conference.

During this preparatory phase, the coordinator’s general responsibilities include the engagement and preparation of the family meeting related to information sharing, relationship building, and ensuring the integrity of the process [20]. The essence of the Family Group Conference is to broaden the circle of care, and that parents of other primary care givers cannot limit these connections or relationships [20]. Invitations to take part in a Family Group Conference are extended to people who care about the young person’s well-being. In addition to the family, it is usually grandparents, uncles, aunts and other family members, friends, acquaintances, neighbours and care providers who take part in the conference. The coordinator does not exclude anyone, unless the family members demonstrate or provide information that a certain individual could be emotionally or physically harmful to other participants or the process. The coordinator ensures that all participants can safely take part in the conference. On average, a conference involves about 12.1 people [19]. The preparation phase does not always lead up to an actual conference. During the research period, 1924 clients were referred to the Family Group Conference Agency. In approximately a quarter of these cases, the preparations for the conference were discontinued [19].

The Family Group Conference itself consists of three phases:

1. The information phase: In this phase, the nature of the problems and the possibilities for support from care providers are discussed. Healthcare professionals are invited to provide information which can support the development of the plan. If the Family Group Conference concerns children who have been placed under supervision, the family guardian involved will present the minimum requirements of the plan during this phase (basic requirements). These requirements always concern the child’s safety.
2. The private phase: The coordinator and healthcare-professionals withdraw to leave the discussion to the network. This discussion results in a plan that everyone agrees with, and that contains agreements, and specifies everyone’s responsibilities. The plan may involve using the capabilities of the network itself as well as requests for assistance from the healthcare professionals. The plan usually combines both sources of support.
3. In the final phase, the family presents the plan to the coordinator. If the child has been placed under supervision, the family guardian also returns to assess the plan. If the plan is safe and legal, it will always be accepted.

The total length of the conference usually varies, of all conferences held in the research period (2011–2012) was 21 % completed within 3 h, 55 % was completed between 3 and 5 h, 23 % was completed between 5 and 8 h and 2 % took more than 8 h [19]. The total process from referral to the actual conference usually takes around six weeks to complete, and the average time investment of the coordinator is 35 h per completed conference.

After the Family Group Conference, the coordinator distributes the plan to every participant who attended the conference, ensuring that every individual who has a role in the implementation of the plan receives the agreement that details responsibilities. Subsequently, the participants of the conference are responsible for the implementation of the plan. Recently, the American Humane Association has developed guidelines for Family Group Decision Making in Child Welfare. In these guidelines it is stated that follow-up meetings, should be part of the process [20]. However, during the research period this was not the case.

### Procedure

In this study, anonymized file data were collected from clients of the Overijssel integrated care system for young people with ID who had taken part in a Family Group Conference (the intervention group) and a comparable group of clients who had not (the control group). The shortened version of the *Zorgpunten Analyse Protocol (ZAP-Kort)* (areas-of-concern analysis protocol) was used to gather standardized information about the present areas of concern in the family and about the aid that was provided for these concerns. These areas of concern were assessed at two moments in time, with a 12-month interval. The usage of care and support from the integrated care system for young people with ID was collected for the entire 12 month period. The first 20 files (17 %) were scored by two researchers in order to obtain consensus on the interpretation of the file data. The remaining files were solely scored by the second author. Our research is carried out in compliance with the Helsinki Declaration and Dutch legislation. Under Dutch law, retrospective file studies are exempted from review by an accredited ethics committee. This kind of research is only subject to the Agreement on Medical Treatment Act, as the data is already available and not collected specifically for research purposes, and the subject does not have to do or abstain from something on behalf of the research. The research protocol is approved by the board of all participating organizations.

### Measurement instruments

ZAP-Kort is an instrument to systematically collect and score information from files [21]. Previous research has shown that this instrument is suitable for detecting improvements in family functioning [5]. The instrument consists of a number of components, which are scored using information obtained from the files, including general information (such as gender and age), healthcare history, areas of concern, and resources used.

The areas of concern are divided into three domains, namely child functioning, family/child-rearing environment and wider environment. These domains and their items were derived from the manual for the Vragenlijst Sociaal Pedagogische Situatie (questionnaire on social and pedagogical situation) [22], a questionnaire developed to support basic diagnostics in child healthcare. The domains involve variables which represent aspects of dysfunctioning. Each domain includes about ten scoring categories, which mostly refer to clearly problematic behaviour and more or less defined environmental factors. In addition, each domain includes a remainder category. Many cases of problematic behaviour and environmental factors were later removed from this remainder category and converted to new scoring categories. The general instruction is that scoring is done based on explicit information from the file. Areas of concern are present (1) or not (0). If no explicit information on a variable is available, or in case of doubt or ambiguity, a 0 is scored. For each domain, a sum-score is also calculated, which presents the total number of areas of concern.

The “resource use” component refers to the care and support which have been used during the research period. This part of the instrument was adapted for the present study. Together with representatives of all participating organisations of the Overijssel integrated care system for young people with ID, we compiled a list of all types of care and support provided by these organisations. Subsequently, standard cost prices for each type of care were determined. These different types of care and support with corresponding cost prices are presented in Table 1. Next, we retrieved the presence and duration of each type of care from the files of the study participants. Finally, costs were calculated by multiplying the number of care units (contacts, hours, months) by their standard cost price.

**Table 1:** Direct medical costs by care or service type

Care or service type	Unit	Cost price
Services local disability support centres		
Information and advice	3,5 h	€ 281,-
Needs assessment	13,5 h	€ 1,043,-
Service coordination	18,5 h	€ 1,444,-
Legal support	15,5 h	€ 1,204,-
Monitoring and evaluation	8,5 h	€ 682,-
Crisis support	10,5 h	€ 883,-
Short-term individual support	22 h	€ 1,685,-
Group activities	7 h	€ 562,-
Diagnostics and treatment		
Diagnostics	1 h	€ 110,-
Treatment	1 h	€ 110,-
Family support services		
Long-term family support (once a week for one year)	1 h	€ 100,-
Intensive family support (twice a week for six months)	1 h	€ 100,-
Families first (four times a week for six weeks)	1 h	€ 115,-
Day care and short-term residential care		
Day care (after school)	4 h	€ 121,-
Day care	1 h	€ 31,-
Short-term residential care (weekend)	1 h	€ 36,-
Long-term residential care		
Residential care (Care Intensity 1)	1 day	€ 140,-
Residential care (Care Intensity 2)	1 day	€ 179,-
Residential care (Care Intensity 3)	1 day	€ 221,-
Residential care (Care Intensity 4)	1 day	€ 259,-
Foster care		
Foster care	1 month	€ 917,-
Child protection		
Family supervision order	1 year	€ 7,571,-
Guardianship order	1 year	€ 5,259,-
Family Group Conference		
Family Group Conference	conference	€ 4,000,-

### Analyses

In addition to a descriptive analysis, t-tests and chi-square tests were used to identify systematic differences between clients who had taken part in a Family Group Conference and those who had not. Changes were then evaluated by calculating the standardized effect size, Cohen’s d. This effect size was calculated by dividing the difference between the pretest and posttest scores by the standard deviation at pretest. According to Lipsey and Wilson (1993), a standardized effect size between 0.56 and 1.2 can be regarded as a large effect; one between 0.33 and 0.55 as a medium-sized effect; and one below 0.33 as a small effect [23].

Subsequently, we calculated the total costs of the formal care and support provided by the integrated care system for young people with ID for both the clients who had taken part in a Family Group Conference and those who had not. Next, we calculated the incremental cost-effectiveness ratio (ICER), which represents the incremental costs (or savings) per area of concern lost in the experimental condition relative to the control condition. Uncertainty was assessed by means non-parametric bootstrapping (5000 times) of the data of individuals participants. The comparison of the simulated ICERs is presented in a cost-utility plane, with differences in costs on the vertical axis and differences in effects on the horizontal axis. If the majority of the estimates appear in the top left-hand quadrant of the plane, the intervention results in a loss of quality of life against additional costs as compared with the control condition, which makes the intervention clearly unacceptable from a cost-effectiveness perspective. If the majority of the bootstrapped ICERs appear in lower right-hand quadrant of the plane, the intervention results in less areas of concern for less costs than the control condition, which makes the intervention clearly superior from a cost-effectiveness perspective. In the other two quadrants the additional costs or savings have to be weighed against the relative changes in areas of concern. The results of the cost-effectiveness analysis are also presented in a cost-effectiveness acceptability curve. The acceptability curve represents the probability that the intervention is cost-effective, given a varying threshold for the willingness to pay for each area of concern lost.

## Results

### Participants

**Fig. 1:**
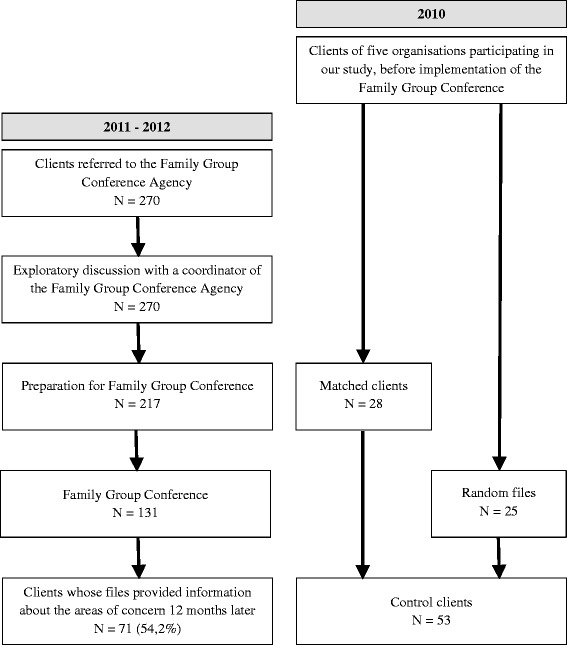
Participants flow through the study

Figure 1 presents the participants flow through the study. In 2011 and 2012, 270 clients were referred to the Family Group Conference Agency by the five participating organisations in the integrated care system for young people with ID in Overijssel. All of them participated in an exploratory discussion with a coordinator of the Family Group Conference Agency. Subsequently, preparations for the actual conference were made for 217 clients. The other clients decided not to participate. The Family Group Conference Agency was able to organize Family Group Conferences for 131 clients (60 %). Reasons for calling off the conference varied from clients opting out, lack of faith in the social network, the absence of a social network willing to participate, and emerging crisis situations.

The intervention group consisted of 71 clients who had taken part in a Family Group Conference and whose files provided enough information to draw conclusions about the effects of Family Group Conferences (54 %). Lack of follow-up data could for the most part be explained by the fact that Family Group Conferences are frequently utilized to prepare a family for the expiration of the Supervision Order, when the youngster turns 18-years-old. As these families usually no longer receive formal care from the integrated care system for young people with ID, there is no follow-up data available.

The control group consisted of the files of 53 comparable clients who had not taken part in a Family Group Conference. This control group included 28 files provided by staff members from the integrated care system for young people with mild ID in the Dutch province of Overijssel, which had been matched for the characteristics of the clients in the intervention group. The remaining 25 files were randomly selected from the client databases of various partners in the integrated care system.

Table 2 presents the characteristics of both groups. Although most families had more than one child, the information about the child always applies to only one of these children, namely the oldest child who was younger than 18 years. The file information in both groups usually related to boys. The average age of the children was between 11 and 12 years, and nearly all of them had been born in the Netherlands, as had their parents. About a third of the children had no parent with ID, while a third had one parent with ID and a third had two parents with ID. The background data did not differ significantly between the two groups.

**Table 2:** Characteristics of the experimental and control groups

	Family Group Conference	Control	*p*-value
Background information			
Number of boys^a^	40 (58 %)	30 (57 %)	0.880
Average age^b^	11.4 (6.3)	12.2 (5.5)	0.431
Born in the Netherlands^a^	69 (96 %)	51 (96 %)	0.477
Mother born in the Netherlands^a^	64 (90 %)	49 (93 %)	0.746
Father born in the Netherlands^a^	64 (90 %)	48 (91 %)	0.694
No parent with ID^a^	23 (32 %)	21 (39 %)	0.317
One parent with ID^a^	20 (28 %)	18 (34 %)	
Both parents with ID^a^	28 (39 %)	14 (26 %)	
Prior history			
Number of years the child has received care^b^	5.5 (4.2)	6.6 (3.9)	0.127
Number of years the family has received care^b^	5.5 (4.8)	6.2 (4.7)	0.447
Number of years the parent(s) have received care^b^	7.8 (5.9)	8.0 (4.9)	0.775
Neglect^a^	20 (28 %)	13 (24 %)	0.650
Sexual abuse^a^	5 (7 %)	5 (9 %)	0.694
Physical abuse^a^	7 (10 %)	8 (15 %)	0.376
Family conflict^a^	27 (38 %)	22 (41 %)	0.695
Divorced parents^a^	25 (35 %)	22 (41 %)	0.475
Areas of concern			
Child functioning^b^	3.4 (2.0)	3.2 (1.8)	0.645
Developmental delay^a^	45 (63 %)	39 (74 %)	0.229
Social and emotional problems^a^	39 (55 %)	33 (62 %)	0.413
Oppositional defiant disorder^a^	24 (34 %)	12 (23 %)	0.176
Attachment problems^a^	18 (25 %)	12 (23 %)	0.727
Aggressive behaviour^a^	13 (18 %)	7 (13 %)	0.445
Family/child-rearing environment^b^	4,0 (2,2)	3,9 (2,3)	0.718
Parent(s) with ID^a^	46 (65 %)	28 (53 %)	0.317
Lack of parenting skills^a^	42 (59 %)	24 (45 %)	0.126
Family conflict^a^	29 (41 %)	14 (26 %)	0.095
Mental health problems of parent(s)^a^	17 (24 %)	15 (28 %)	0.842
Lack of understanding of problems^a^	14 (20 %)	16 (30 %)	0.178
Wider environment^b^	1.5 (1.3)	1.1 (1.2)	0.130
Financial problems^a^	20 (28 %)	20 (38 %)	0.260
Problems in the residential environment^a^	20 (28 %)	8 (15 %)	0.085
Lack of social netwerk^a^	10 (14 %)	4 (8 %)	0.255

^a^Absolute numbers and percentages ^b^Averages and standard deviations

The history before the start of the study was also comparable between the groups. The families had been receiving professional care for years. In addition, many of the parents had already been receiving professional support for personal issues. About a quarter of the children had a prior history of neglect, while slightly more than 10 % of the children had been sexually abused and more than 10 % had a history of physical abuse. About 40 % of the families had a history of family conflict, and roughly the same percentage of the parents was divorced. No significant differences were found between the two groups as regards prior history.

Finally, the groups did not differ significantly in the average number of areas of concern identified at pretest for the three ZAP-Kort domains. On average, there were three areas of concern from the child-functioning domain. The most common areas of concern in this domain were a developmental delay, social and emotional problems, Oppositional Defiant Disorder, problems of attachment, and aggressive behaviour. Within the domain of family/child-rearing environment, there were on average four areas of concern, the most common ones being the consequences of one or both parents’ ID, lack of parenting skills, family conflict, mental health problems of one or both parents and lack of understanding of problems (often combined with resistance to aid). The fewest areas of concern were found for the wider environment domain, with an average of just over one area of concern. The most common areas of concern for this domain were financial problems, problems in the residential environment and lack of social network.

### Effects of the Family Group Conference

Table 3 presents the improvement over time in both groups. All three domains of the areas of concern analysis showed a significant improvement in the intervention group compared to the control group. Child functioning improved in the intervention group from 3.35 to 2.28 areas of concern (Cohen’s d = 0.55), while there was hardly any improvement in the control group (Cohen’s d = 0.04). Both the absolute difference in numbers of areas of concern at posttest and the difference in the amount of change were statistically significant (*p* < 0.05). The number of areas of concern within the family/child-rearing environment decreased from 4.01 to 2.04 in the intervention group (Cohen’s d = 0.89), while the number of areas in the control group decreased from 3.87 to 3.42 (Cohen’s d = 0.20). Again, both the absolute difference in the number of areas of concern at posttest and the difference in the amount of change were statistically significant (*p* < 0.05). Areas of concern from the wider environment domain showed a decrease from 1.46 to 0.66 in the intervention group (Cohen’s d = 0.63), compared to a decrease from 1.11 to 0.81 (Cohen’s d = 0.24) in the control group. While the absolute difference in the number of areas of concern was not statistically significant, the difference in the amount of change was.

**Table 3:** Areas of concern; averages and standard deviations

Areas of concern	Group	Pretest	Posttest	Difference	Effect size
Child functioning	Intervention	3.35 (2.04)	2.28 (1.73)	1.07 (1.59)	0.55 (0.82)
	Control	3.19 (1.82)	3.11 (1.72)	0.08 (1.02)	0.04 (0.52)
Child-rearing environment	Intervention	4.01 (2.20)	2.04 (2.04)	1.97 (2.04)	0.89 (0.92)
	Control	3.87 (2.25)	3.42 (2.49)	0.45 (1.59)	0.20 (0.72)
Wider environment	Intervention	1.46 (1.31)	0.66 (0.79)	0.80 (1.25)	0.63 (0.98)
	Control	1.11 (1.22)	0.81 (1.00)	0.30 (0.89)	0.24 (0.70)
Total	Intervention	8.83 (3.18)	4.99 (3.14)	3.85 (3.08)	1.17 (0.94)
	Control	8.17 (3.40)	7.34 (3.19)	0.83 (2.31)	0.25 (0.70)

As regards the total number of areas of concern, the average decrease in the intervention group was 3.85, while the control group showed a decrease of 0.83 areas of concern. The standardized effect size in the intervention group was 1.17, which corresponds to a large effect. The standardized effect size in the control group was 0.25, corresponding to a small effect.

### Cost-effectiveness

Table 4 presents the average annual costs of the care and services provided by the integrated care system for young people with mild ID. Without the additional costs for the Family Group Conferences (€ 4000), both groups did not significantly differ. However, when the additional costs of the Family Group Conferences are included, the average annual costs of the intervention group are significantly higher than the average annual costs of the control group. Therefore, Family Group Conferences did not result in decreases in formal care.

**Table 4:** Annual costs of the integrated care system for young people with ID

Care or service type	Family Group Conference group	Control group
Services Local disability support centres	€ 811 (1,572)	€ 469 (1,340)
Diagnostics and treatment	€ 566 (2,412)	€ 338 (957)
Family support services	€ 4,005 (4,908)	€ 1,404 (3,376)
Day care and short-term residential care	€ 1,632 (6,537)	€ 4,606 (13,167)
Long-term residential care	€ 18,655 (29,094)	€ 13,457 (28,363)
Foster care	€ 1,718 (3,364)	€ 5,052 (5,489)
Child protection	€ 6,291 (2,858)	€ 5,782 (3,062)
Total costs formal care	€ 33,680 (27,313)	€ 31,107 (31,106)
Family Group Conference	€ 4,000 (0)	-
Total costs	€ 37,680 (27,313)	€ 31,107 (31,106)

**Fig. 2:**
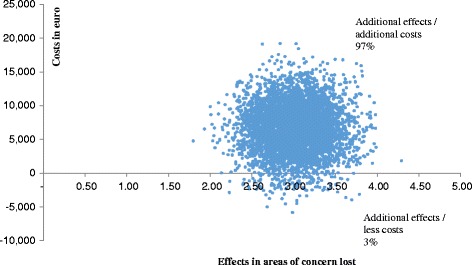
Cost-effectiveness plane. Each dot (*n* = 5000) represents a bootstrapped ICER

The incremental cost-effectiveness ratio (ICER) was calculated as the difference in costs (€ 6573) divided by the difference in effects (3 areas of concern). This means that for the loss of each area of concern by offering a Family Group Conference, the additional costs amount to € 2180. Bootstrapping of the data of the individual participants yields an median ICER of € 2197 (95 % Confidence Interval: -€ 1951 - € 4639). The ICER is surrounded by a certain amount of uncertainty, which is presented in the cost-effectiveness plane (Fig. 2). Each dot of the cost-effectiveness plane represents a bootstrap replication (*n* = 5000) of the ICER; the majority of the dots (97 %) are in the upper right-hand quadrant, indicating a 97 % probability that the Family Group Conference generates better effects against higher costs. The remaining dots (3 %) are in the lower right-hand quadrant, indicating a 3 % probability that the Family Group Conference generates better effects while saving money.

**Fig. 3:**
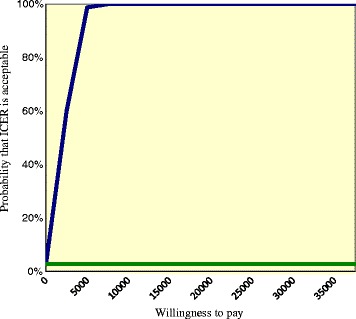
ICER acceptability curve. Probability the Family Group Conference is acceptable given varying thresholds for willingness to pay

The acceptability curve is presented in Fig. 3. The Family Groups Conference has a probability of 3 % of being more acceptable than the comparator condition from a cost-effectiveness point of view under the conservative scenario that there is no willingness to pay for the loss of areas of concern. However, people are generally willing to pay for health benefits. When the willingness to pay is raised to € 2500 per area of concern lost, the Family Group Conference has a probability of 60 % of being cost-effective compared to the control condition. Finally, when the willingness to pay is raised to € 5000 per area of concern lost, there is a 99 % probability of cost-effectiveness.

## Discussion

### Main findings

The problems in the group of clients of an integrated care system for young people with ID who had taken part in a Family Group Conference greatly decreased over a period of twelve months. This decrease in the number of areas of concern was seen in all three domains examined: child functioning, family/child-rearing environment, and wider environment. There was a much smaller decrease in the number of problems in the group that had not taken part in a Family Group Conference. These results suggest that this intervention is effective. The Family Group Conference did not result in a decrease of the usage of formal care by the integrated care system for young people with ID. Or at least, not in the short-run.

### Strengths and weaknesses of the study

It is important to consider both the strengths and weaknesses of the current study when interpreting the above-mentioned results.

The main limitation concerns the way in which the study groups were assembled. The strongest proof of an intervention’s effect is provided by studies in which research groups are randomly composed and where participants are randomly assigned to either the experimental or control group. Random assignment was not possible in the current study, which reduces the evidential value of the findings. Since the research groups had not been randomly assigned, there was an increased risk that the groups were not entirely comparable. Even though no significant pretest differences between the groups were found, it is possible that they differed in areas we did not assess.

Another limitation concerns the sample size in general and the size of the control group in particular. The control group was markedly smaller than the intervention group (53 versus 71). Although we did attempt to involve a bigger sample, this effort was only partly successful. In both the experimental and control group, it proved to be difficult to gather sufficient information from the files to monitor the progress of the clients, which made it impossible to report on all clients. The control group may have been too small to enable us to identify all possible pretest differences with the intervention group. On the other hand, the study group did prove to be large enough to reveal significant differences at posttest, which implies that differences at pretest were probably not very large.

Our data collection strategy could have biased results, because data for the intervention and control group were collected at different points in time. Practices and contexts change over time, which could influence results. However, we believe that the selected procedure was the best available option. Control group data originated from the year before the introduction of the Family Group Conference in the integrated care system for young people with ID in the Dutch Province of Overijssel. Although practices change of time, this is usually a long-term process. The scored file data appeared similar: intervention and control families displayed similar problems and utilized similar resources.

Another disadvantage of our research method is that retrospective research can only use information actually included in the files. If information is missing, there are few options open to collect it. The result was a smaller selection of clients than had been intended. Excluding clients with missing file data may have had some consequences for the representativeness of the sample. Unfortunately, we cannot conclude whether this is the case, since file data from clients was excluded because of insufficient data was not scored. In other words, it is not possible to compare clients who were selected for the study with those who were excluded.

In our study, we have based our conclusions on the results of the Family Group Conference on file data instead of on self-reported changes by the participants. This evaluation method might be considered as unconventional as the intervention in itself is highly participative. However, we believe that in our particular sample the perspective of the social workers is presumably more reliable than self-reports of the included children or parents. One of the problems in the integrated care system for young people with ID, is that the magnitude of the problems in not always acknowledged by the family. People with ID find it difficult to recall earlier events to analyse and reflect on them [24]. Lack of understanding of the problems was one of the most prevalent areas of concern in the family environment. The majority of the children in our sample was under supervision, because the child’s safety and wellbeing was considered at risk. The family guardian is legally authorized to oversee that the problems threatening the child’s development are resolved. The perspective of the family guardian and other social workers involved with family, is therefore a very valuable and reliable measure of the achieved progress. In addition, one of the reasons to conduct this study was that social workers were very sceptical about the applicability of the FGC in the integrated care system for young people with ID. The fact that the conclusion that FGCs improved the results of the formal care, makes our results even more convincing.

Despite the study’s weaknesses, its results provide useful new information for researchers and professionals. While there have been a great number of studies on the Family Group Conference approach in recent years, the number of controlled studies remains limited. Thus, any new controlled study makes an important contribution to the evidence base for using the Family Group Conference approach in healthcare. The present study was, at the best of our knowledge, even the first controlled study to examine the effects on the problems of children and/or parents with ID. Our findings reveal that these families can indeed also benefit from this approach, something which had been previously doubted.

Our analysis of both effects and costs of the Family Group Conferences in the integrated care system for young people with ID also contributes to the debate on the purpose and position of Family Group Decision Making in the formal health care and welfare system. Family Group Conferences should not be considered as a cheaper substitute for formal care. Several other studies point out that without careful preparations and sufficient follow-up, positive effects will diminish [25, 26]. Family Group Conferences should be viewed as a step in the ongoing process of collaboration and empowerment in which families are invited and supported. Our findings suggests that Family Group Conferences promote the integration of both formal and informal care systems.

Of course, the current study does not answer all questions, and additional research within the integrated care system for young people with ID remains necessary. Our findings suggest that children and/or parents with ID can also achieve improvements with the help of a Family Group Conference. It is unclear whether this is the case for the entire target group, which consists of both children with ID and children of parents with ID. It seems likely that the presence of one or both parents with ID could influence the effectiveness of a Family Group Conference. However, our sample size was too small to draw any conclusions about various subgroups.

In addition, it is unclear how representative our study sample was for the whole target group. During the research period, 270 families were referred to the Family Group Conference Agency. Subsequently, preparations for the actual conference were made for 217 clients. In the end, 131 clients participated in a Family Group Conference. Unfortunately, as we were not able to assess the file data of the clients not participating in a conference, it is unclear whether the areas of concern differ between clients willing to participate and those declining participation. At the very least it can be assumed that a long-lasting history of healthcare use and relatively severe problems should form no obstacle to achieving a positive effect with a Family Group Conference. On average, the families in our sample had already received 5.5 years of professional family support before a Family Group Conference was held, and this healthcare trajectory was even longer if the aid parents received for personal problems is also taken into consideration: in that case, the trajectory had lasted an average of eight years. In addition, a large proportion of the families in our research group were characterized by relatively severe issues: at the start of the study, the family average was 8.55 areas of concern. In another study, using the same instrument to measure the effects of the Family Group Conference in youth protection, the pretest number of areas of concern was 5.83 [5]. In any case, additional research remains necessary to determine whether the results presented here can be generalized to the entire population of children with ID and children of parents with ID, and whether the decrease in the number of areas of concern is a long-term effect.

Finally, it might be helpful to weigh in mind the context in which research on Family Group Conferences is done. Researching the effectiveness of Family Group Conferences is challenging. Multiple attempts to rigorously test its effectiveness in randomized controlled trials have failed because of the challenges of recruiting families into the study, and several other controlled studies lack equivalent control groups [26]. The Family Group Conference is a decision-making model, which can be applied in numerous situations to a variety of problems. Successful conferences result in a plan, specific to a particular context and situation. Therefore, it is extremely difficult to decide in advance what the preferred outcome should be [26]. In addition, the preferred outcomes may differ among informants. Family Group Conferences are implemented in different care systems, but are mostly directed towards vulnerable families with histories of parenting problems, neglect and even abuse. Often, there is a lack of trust between family members among themselves, and between family members and the formal care system, especially when a Family Supervision Order is in place. These circumstances complicate Family Group Conferences research. According to Burford [27], research into the results of Family Group Conferences should evaluate the safety and wellbeing of the main participant, as well as changes in the services around families. Family Group Conferences should not only be a vehicle to accommodate vulnerable families to society. At the same time, social and health care services could also be influenced by the needs of these families. Our study, like most available controlled studies, focused merely on the safety and wellbeing of children and/or parents with ID. In addition, utilization of social and health care services was considered. Although our results suggest an interplay between both formal and informal care, we did not evaluate whether the attitude of formal service providers was changed by the participation of the extended family. This perspective should be considered in future research.

## Conclusions

Our findings reveal that people with ID can also benefit from Family Group Conferences, something which had been previously doubted. Support from the social network, however, does not substitute formal care. Family Group Conferences appear to lead to better organized support around the family with informal and formal care joining forces, instead of frustrating each other which is unfortunately often the case.
